# Limitation of therapeutic effort in pychiatric patients. about a case

**DOI:** 10.1192/j.eurpsy.2021.1000

**Published:** 2021-08-13

**Authors:** C. Martín Villarroel, L. Carpio Garcia, J. Dominguez Cutanda, G. Belmonte García, J. Matsuura, M. Sánchez Revuelta, M. Fernández-Torija Daza, E. García

**Affiliations:** Psiquiatría, Complejo Hospitalario Universitario de Toledo, Toledo, Spain

**Keywords:** autonomy, limit the therapeutic effort, ethical, quality of life

## Abstract

**Introduction:**

Thanks to advances in medicine, more diseases are being cured, but this benefit can become a problem when it causes a worsening of quality of life.

**Objectives:**

The objective of this paper is to analyze, regarding the following case, the convenience of treating or to limit the therapeutic effort (LTE) in psychiatric patients who are in situations at the end of life.

**Methods:**

62-year-old woman begins with depressive symptoms from financial problems. In 4 months ago she makes four suicide attempts (drug overdose, cuts, self-stabbing, and precipitation), being hospitalized in ICU after latter because of multiple trauma and shock. During that time, she had a bad evolution with several complications that made LTE be evaluated. A bibliographic search was performed from different database (Pubmed, TripDatabase) about LTE and ethical implications.

**Results:**

Trying to prolong life by disproportionate means in a patient with a poor prognosis or poor quality of life is bad practice. We must assess the severity, quality of life, capacity and preferences of the patient to decide to treat or not, thus guaranteeing the principle of beneficence. It is also important to respect the principle of autonomy, accepting patients can refuse treatment. All this is equally applicable to psychiatric patients, whom we should not stigmatize but rather evaluate their ability to decide, as in any person.

**Conclusions:**

In conclusion, in situations of high suffering and near death, it is necessary a complete evaluation of the patient (psychiatric or not) is carried out in order to act in the most ethical way.

